# Development, Challenges, and Evolution of the Log2Lose Intervention for Weight Management: Randomized Controlled Digital Health Trial

**DOI:** 10.2196/70842

**Published:** 2025-11-17

**Authors:** Ryan Jeffrey Shaw, Hailey Miller, Cherie Barnes, Clemontina Davenport, Sarah Morton-Oswald, Sarah Jackson, Michelle Bean, Jeff Cohen, Chance Griffin, Jane Pendergast, Maren Olsen, Jennifer Gierisch, Corrine Voils

**Affiliations:** 1 School of Nursing Duke University Durham, NC United States; 2 School of Nursing Johns Hopkins University Baltimore, MD United States; 3 Department of Biostatistics and Data Science Wake Forest University Winston-Salem, NC United States; 4 Department of Biostatistics & Bioinformatics Duke University School of Medicine Duke University Durham, NC United States; 5 Department of Population Health Sciences Duke University School of Medicine Duke University Durham, NC United States; 6 Purple Workshops Chicago, IL United States; 7 Duke University Durham VA Health Care System Durham, NC United States; 8 Division of Epidemiology Department of Internal Medicine University of Utah Salt Lake City, UT United States; 9 William S Middleton Memorial Veterans Hospital Madison, WI United States

**Keywords:** weight loss, weight maintenance, diet, mobile phone, remote monitoring

## Abstract

**Background:**

Long-term adherence to weight loss behaviors is challenging, as most individuals who achieve significant weight loss regain 1-2 kg per year. Financial incentives can reinforce weight-loss initiation and maintenance behaviors, but optimal strategies remain unclear.

**Objective:**

This paper describes the design, technical architecture, and operational workflow of Log2Lose, a 5-year, multisite randomized controlled trial testing different financial incentive strategies to promote weight loss and maintenance. We detail the platform’s integration with external devices, automated data collection, and adaptations to maintain intervention fidelity in the context of evolving technology and regulatory requirements.

**Methods:**

The Log2Lose platform collects daily weight and dietary data from cellular scales and fitness tracking applications, calculates weekly incentive eligibility, and sends automated feedback and motivational text messages. We summarize the technical adaptations, message delivery performance, data completeness, and the balance between automation and manual support required to ensure data integrity.

**Results:**

By the end of the study, 706 participants recorded 181,285 weights and 114,144 daily calorie entries. The platform sent 126,283 text messages and calculated 35,187 incentive payments, with 99.4% (34,976/35,187) processed automatically. Adaptations addressed device integration changes, application programming interface discontinuations, and new text messaging regulations. Despite automation, ongoing technical support was essential for resolving delivery errors, device issues, and data anomalies.

**Conclusions:**

Log2Lose demonstrated that large-scale, fully remote weight loss interventions can be implemented using consumer technology paired with a robust, adaptable platform. Success depended on flexible software design, continuous monitoring, and responsive technical support to navigate regulatory and technological changes. Log2Lose offers a practical model for processing remotely collected longitudinal dietary and weight data, providing valuable guidance for researchers, health care providers, and employers developing similar digital health interventions.

**Trial Registration:**

ClinicalTrials.gov NCT04770909; https://clinicaltrials.gov/study/NCT04770909

## Introduction

Over 40% of US adults have obesity [1], increasing the risks for diabetes, hypertension, and higher medical costs [2]. Although numerous weight loss programs achieve an average weight loss of at least 5%, long-term adherence remains a challenge, with most individuals regaining 1-2 kg per year [3].

Financial incentives can reinforce weight-loss initiation and maintenance behaviors, but optimal strategies remain unclear. Although evidence suggests that incentivizing both weight loss and dietary self-monitoring may be effective, it is unknown whether long-term outcomes are best supported by reinforcing the outcome of interim weight loss, the behavior of dietary self-monitoring, or both [4]. In addition, most prior studies distributed rewards at the end of the program rather than as weight loss occurs [5-10]. Incentive timing may influence outcomes, as continuous reinforcement can accelerate behavior acquisition, whereas intermittent reinforcement can sustain it [11]. Little is known about how to structure and implement incentives in remote interventions to maintain adherence to weight loss behaviors over time [12,13].

To address these gaps, we launched Log2Lose, a 5-year, multisite randomized controlled clinical trial in September 2020, to test whether reinforcing interim weight loss, dietary logging, or both improves the proportion of people who achieve clinically significant weight loss over 78 weeks (18 months) relative to a control group with no incentives.

Originally designed to include in-person group meetings, telephone calls, incentives, text messaging, and in-person data collection, the trial pivoted to fully remote delivery during the COVID-19 pandemic. Since launch, the technology landscape has evolved, requiring adaptations to account for changes in ownership, business agreements, and consumer-protection policies among technology providers. In March 2023, the US Federal Communications Commission implemented its first consumer protection rules targeting text messaging [14], requiring mobile providers to block certain messages deemed likely to be illegal or harmful. Concurrently, mobile service providers deployed software to prevent spam- and fraud-prevention software, increasing the risk that legitimate intervention messages would be flagged as spam or “fail to send” if classified as potentially unwanted or high-risk (eg, high-risk financial services, debt collection/forgiveness, “get-rich-quick” schemes) [15].

The primary objective of this study is to describe the Log2Lose intervention’s technical architecture, integration with external devices, and operational workflows, including automated and manual procedures for data monitoring and incentive processing. We detail adaptations made to maintain intervention fidelity amid regulatory and technological changes—which can be expected in a 5-year trial—and we present operational outcomes, including message delivery rates, device data completeness, and weight measurement patterns, along with lessons learned for designing scalable, technology-supported weight-loss interventions.

## Methods

### Trial Design

This study’s primary objective was to assess the efficacy of financial incentive strategies for achieving person-level loss of ≥5% of baseline weight. The trial spanned three 6-month phases: phase I, incentivized initiation with 13 biweekly group sessions; phase II, incentivized maintenance with 3 monthly group sessions plus 5 monthly individual calls; and phase III, a 26-week, nonincentivized maintenance program with 3 bimonthly calls. Group sessions and calls were led by a registered dietitian.

### Participants

Participants with obesity were recruited in Madison, WI, and Durham, NC, USA. The inclusion and exclusion criteria are detailed in ClinicalTrials.gov NCT04770909 and published [16]. Related to the Log2Lose technology, participants met requirements if they were able to speak and read English; able to download and use the Fitbit and MyFitnessPal smartphone apps daily; owned a smartphone with a data and texting plan; had reliable internet access; had the ability to participate in video calls via a smartphone, tablet, or computer; and as described below, later in the trial agreed to create a Gmail account after Google acquired Fitbit.

### Interventions

Participants were randomized to one of the 4 arms: incentives for both dietary self-monitoring and weight loss, with partial incentives if only 1 criterion was met (arm 1, combined); incentives for dietary self-monitoring only (arm 2, diet only); incentives for weight loss only (arm 3, weight only); or no financial incentives (arm 4, control) [16].

All participants received a BodyTrace cellular scale and were encouraged to weigh themselves regularly: twice weekly in phase I (weight loss) and once weekly in phase II (maintenance). Participants were instructed to keep the scale on a stable surface and restrict use by others. Participants were asked to track daily food and beverage by using a smartphone app and at 6-month intervals, to wear an activity tracker for 7 days.

All participants received automated motivational text messages twice weekly for 78 weeks (phases I-III), reinforcing educational themes and behavioral skills from group sessions and counseling calls. In phases I and II, participants in incentivized arms received weekly text messages indicating whether they had earned an incentive. Messages were sent between approximately 8 AM and 11 AM local time, with no adjustment for travel across time zones. Due to the COVID-19 pandemic, remote weights replaced in-person measurements as the primary outcome. Prior research supports the validity of remote monitoring compared with in-person weighing, with the added convenience of avoiding travel [17].

Control-arm participants received the same devices and platform access but no financial incentives. All participants received the same frequency and timing of motivational text messages, which were standardized prompts rather than tailored feedback. No additional behavioral counseling was provided via the platform.

### Outcomes

This study reports the outcomes for the total number of text messages received by day of the week and message type, the delivery success rate, the total number of weights transmitted, the average number of daily weigh-ins per participant, and the total number of calorie entries received. It summarizes the number of incentive payments processed automatically by the software compared to those processed manually. Finally, we report common technical and troubleshooting examples.

### Sample Size and Randomization

Participants were randomized equally to one of the 4 arms by using a computer-generated block randomization sequence accessible only by the study statisticians. Participants were blinded to the incentive criterion of other arms. Sample size and power calculations were conducted using the methodology for multi-arm tests of proportions in PASS 2019. Further details are available in the published protocol paper [16].

### Ethical Considerations

Study conduct was overseen by the University of Wisconsin-Madison institutional review board (approval 2020-1693) and a Data Safety Monitoring Board that met at least annually throughout the study period for ethics review and approval. All participants provided informed consent and could opt out of the trial at any time. People completed a web-based prescreening survey, followed by telephone screening and then final in-person eligibility screening and informed consent. Data were stored in secure password-protected databases only accessible to institutional review board–approved study staff. Compensation was provided for assessment completion at weeks 26 (US $40), 52 (US $25), and 78 (US $35).

### Platform Description

The Log2Lose platform was developed to automate data collection, incentive processing, and monitoring procedures in support of the randomized trial. The system integrated weight and dietary data from consumer devices and applications, applied prespecified algorithms to determine incentive eligibility, and delivered feedback to participants via text messages. Research staff had access to a dashboard that allowed monitoring of data completeness, device connectivity, and message delivery. These features were designed to maintain intervention fidelity and to provide safeguards against data anomalies or technical failures. Importantly, the platform was developed to be adaptable, enabling adjustments to accommodate vendor-driven changes such as the discontinuation of the MyFitnessPal–Fitbit application programming interface (API) and shifting the US Federal Communications Commission text messaging regulations, which might otherwise have compromised study integrity.

**Figure 1:**
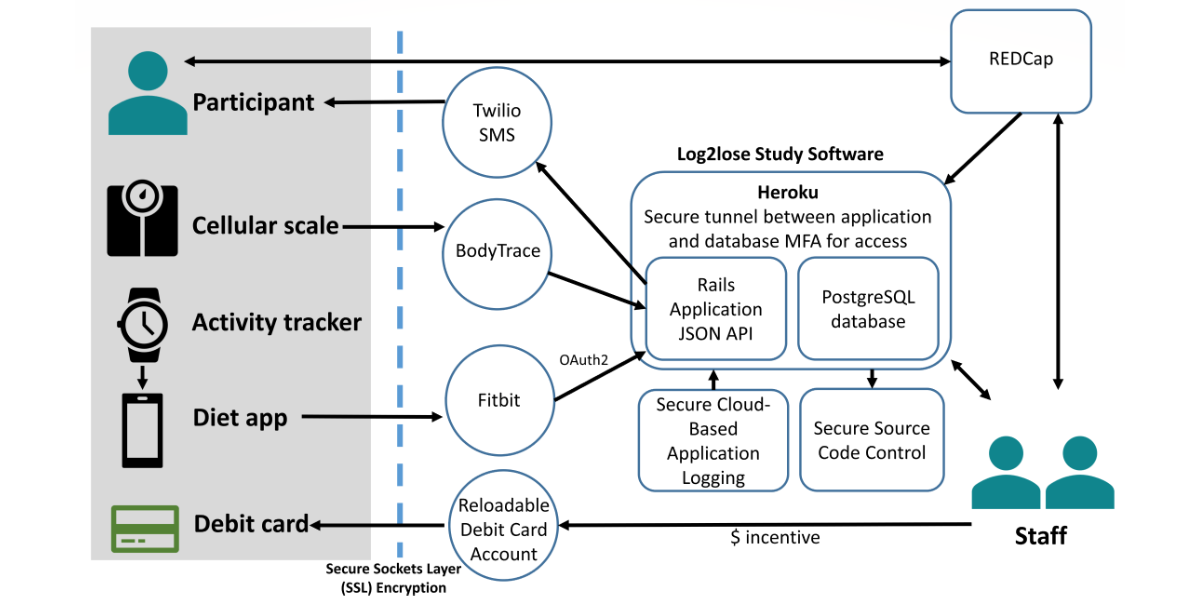
Overview of the study software infrastructure, which securely integrates data from participant devices (cellular scale, activity tracker, diet app) and incentive payments with study software using a web API. The software operated on a Heroku platform-as-a-service cloud server, utilizing a Ruby on Rails application linked to a PostgreSQL database. Data are transferred via secure protocols to a central database, enabling automated messaging, incentive processing, and synchronization with REDCap for staff oversight. API: application programming interface; MFA: multi-factor authentication; REDCap: Research Electronic Data Capture.

The platform applied prescheduled weekly algorithms to determine incentive eligibility (Figure 1). It operated on a Heroku platform-as-a-service cloud server, utilizing a Ruby on Rails application linked to a PostgreSQL database. Data from BodyTrace scales (weights), Fitbit activity trackers (steps per day, minutes active), and Fitbit and MyFitnessPal dietary apps (calories logged per day) were securely imported into the platform using a web API. The BodyTrace scale measured weight and had an embedded cellular chip using the global system for the mobile communications network, which transmitted weight data to the BodyTrace servers. Each scale’s international mobile equipment identity number was recorded at enrollment, linked to the participant ID, and used to retrieve data via API.

During enrollment, participants created a Fitbit account and received an authorization link from the platform. Upon authorization, the platform linked the Fitbit account to the participant ID, enabling import of daily calories, steps, and activity minutes via API. MyFitnessPal did not offer an API to pull data into the platform. Therefore, participants also linked their Fitbit account to the MyFitnessPal app so that calorie data from MyFitnessPal would transfer over to Fitbit.

Participant information (eg, enrollment, surveys) from the Research Electronic Data Capture (REDCap) system, a secure online platform for data capture and storage, was integrated into Log2Lose via API for analysis and study management [18,19]. This integration also included automated notifications for participants and staff.

### Log2Lose Platform

#### User Interface

The platform used a messaging API to display study metrics on interactive dashboards (Multimedia Appendices 1 and 2), including participant data, message counts, delivery rates, and text message errors. Additional dashboard features included calorie counts, weights, incentive earnings, participant demographics, Fitbit connection status, and scale battery levels, with all data downloadable. The platform (1) provided interactive dashboards for research staff; (2) generated comprehensive summaries of calorie entries, weigh-ins, incentives, and text message statistics, including detailed views of failed messages; and (3) displayed participant information by study site and cohort, Fitbit connection status, scale signal, and battery status. These features supported operational and safety decisions such as verifying anomalous weights, resolving data integration issues, adjudicating incentive eligibility in edge cases (eg, device outages), and triggering participant outreach when adherence abruptly declined. Decisions were made by trained research coordinators in accordance with the study protocol, with escalation to the study clinician for safety-related concerns. Many platform features were added iteratively in response to emerging operational needs. Figures S1A and S1B in Multimedia Appendix 1 provide detailed views of the dashboards.

The platform allowed filtering of participant data by cohort or site and provided individual profiles displaying study details, timestamps, carrier information, and recent Fitbit and BodyTrace data. Links to full historical records are available from each profile (Multimedia Appendix 2).

#### Incentive Algorithms, Processing, and Incentive Text Messages

Each week, the platform determined whether participants qualified for incentives for dietary self-monitoring, weight loss, or both, based on data from the participant’s cellular scale and fitness application, their randomized study arm, and their position in the study timeline (Multimedia Appendix 1).

This payment algorithm was embedded within the Log2Lose software (hosted on GitHub with secure cloud-based application logging), which generated weekly incentive reports identifying eligible participants. Payments were processed in near real time by a research team member and loaded onto study-provided debit cards.

Participants were notified of their weekly incentive status via Twilio text messages sent on Mondays; if Monday was a holiday, messages were sent the following day. The delivery rate was calculated as the number of messages accepted by the downstream service (carrier for SMS text messages; provider API for push/email) divided by the number attempted by Log2Lose. The success rate was calculated as the proportion of delivered messages that received a positive delivery receipt from the downstream service. Message opens were not tracked (see Limitations).

### Data Quality and Monitoring

#### Automated Procedures

Because errant weights were expected from study-provided scales, the platform automatically screened and flagged measurements showing implausible deviations (>3%) from the most recent accepted weight. Such deviations may have resulted from another person using the scale or user error, such as not calibrating the scale prior to use. Participants were also asked to weight twice in close succession if the first weight seemed unreasonable. For incentive calculations, the measurements were labeled as rejected, and they were not considered in determining whether an incentive was earned. The system also monitored syncing issues with retrieving dietary data.

#### Manual Procedures

The statistical team conducted regular checks to detect patterns of repeated rejected weights and notified research coordinators for manual review. Several situations may prompt such review. For example, if a long time had passed since a participant last used the scale, their weight may fall outside the 3% threshold from their previous accepted measurement. In these cases, the participant was contacted, their weight verified, and the last accepted weight was manually reset. Another common scenario occurred when another household member used the scale, generating a weight outside the threshold. Additionally, participants may contact a study coordinator if they believed they incorrectly did not receive an incentive. The coordinator then reviewed the data to determine whether a correction was needed—usually for an unusual situation. If any changes were made, the coordinator received approval from the principal investigators. Such corrections were rare.

### Statistical Analyses

We summarized the total number of messages received by day of the week and by type of message (incentive or motivational), as well as the delivery success rate. We also summarized the total number of weights transmitted, the average number of daily weigh-ins per participant, and the total number of calorie entries received. We described the number of incentive payments processed automatically by the software versus those processed manually. Descriptive statistics were used to summarize study participation, message distribution, weight recordings, calorie entries, and incentive payments. Frequencies and percentages were calculated for categorical outcomes, including message type, delivery success, and incentive processing mode, while means, standard deviations, and ranges were used for continuous outcomes such as daily message counts, number of weight recordings per participant, and calorie entries. Temporal patterns in logging behavior were described narratively in relation to technical disruptions, and rare events such as delivery failures and manual incentive processing were reported descriptively given their low frequency.

## Results

A total of 706 participants were randomized and provided a baseline weight—348 and 358 across Wisconsin and North Carolina, respectively. Final data collection was completed in April 2025.

### Text Messaging

By the end of this study, 126,283 messages were sent, with a daily maximum of 1414. Message distribution was balanced between sites: 62,276 to 348 participants in Wisconsin and 64,007 to 357 participants in North Carolina. One participant withdrew before receiving the first message; thus, 705 participants received messages.

Incentive messages were sent primarily on Mondays (n=26,111) and to a lesser extent on Tuesdays (n=300). Motivational messages were sent on Tuesdays (n=50,352) and Fridays (n=49,319). An additional 201 messages were sent manually on a Wednesday to report a technical error.

Overall, >99% of the messages were delivered successfully. Delivery failures typically resulted from international travel, prolonged service loss, or extended periods with phones turned off. The most common failure type involved messages flagged as spam by mobile carriers despite Twilio’s trusted-source agreements. Possible triggers included absence of sender identification, use of dollar signs or shorthand characters, excessive exclamation marks, and repetitive content, although carriers did not disclose specific criteria.

### Weights

By study end, 706 participants recorded a total of 181,285 weight measurements from the cellular scales. Of these, 94.9% (172,096/181,285) were accepted. The highest number of weights transmitted in a single day was 349. On weighing days, participants recorded an average of 1.24 (SD 0.316) measurements, reflecting multiple consecutive weigh-ins.

### Calorie Data

There were 114,144 daily calorie entries from 694 participants, with a single day maximum of 256. Logging declined sharply around the 90-day mark, partly due to MyFitnessPal–Fitbit API issues in December 2021. Initially, Fitbit data were retrieved via MyFitnessPal for cohort 1, but persistent API problems prompted a switch to Fitbit-only logging. This improved reliability by removing third-party syncing, though participants reported lower satisfaction with Fitbit’s interface. Google’s acquisition of Fitbit required all users to have Google accounts by the end of 2025. In July 2024, Fitbit consolidated its dashboard into the mobile app, ending web access and affecting our final cohort.

### Incentives

**Figure 2:**
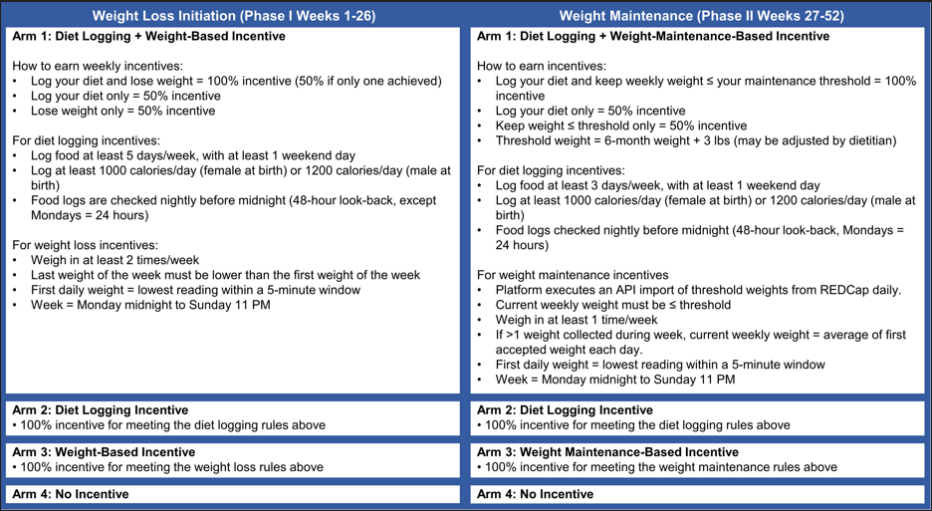
Incentive algorithms. Participants with obesity were randomized to one of four incentive algorithms, which were applied to data collected from a diet app, a weight scale, both, or neither across two study phases. Phase I (weeks 1–26) focused on weight loss, providing rewards for diet logging, weight loss, or both. Phase II (weeks 27–52) emphasized weight maintenance within a defined threshold while continuing diet logging. Algorithm rules specified the required logging frequency, calorie minimums, and weigh-in schedules. The four study arms were: combined incentives, diet-only, weight-only, and no-incentive control. API: application programming interface; REDCap: Research Electronic Data Capture.

The platform automatically calculated and queued 35,187 incentives during phases I and II. Only 222 incentives (0.6%) across both sites required manual processing, mainly due to technical or behavioral complexities (Figure 2; Table 1).

**Table 1:** Platform and technical troubleshooting examples.

Issue type	Example	Potential problems	Solutions
Text message delivery	Monitoring for delivery failures	Spam flagging Participant opted out Phone off/out of service Landline number given Daylight savings errors	Automate resends Investigate delivery failures Confirm participant preferences
Weight data	Missing or inaccurate data	Incorrect scale ID entry Scale out of carrier range Low battery Wrong scale used Scale not tared correctly Weighing fully clothed Scale not on a level surface Household member stepping on scale	Verify scale ID Verify participant stepping on scale Support scale functionality and placement Ensure battery power
Dietary data	Logged food does not appear in the platform	Outdated device operating system Old Fitbit app version Multiple Fitbit accounts Enterprise Google email on device Missed authorizations Technical bugs with Fitbit app Accidental revocation of authorization Data transmission issues	Update device and Fitbit app Ensure single account use Check authorization and data transmission Manually sync foods Reauthorize and send new code
Dietary application API^a^	MyFitnessPal dietary data not retrievable	API connection cut by MyFitnessPal	Shift to Fitbit dietary app
Incentives	Participant reports nonreceipt	Scale misuse Weight discrepancies Scale shared with others Scale out of carrier range Weighing outside incentive period Time zone issues Insufficient weigh-ins Multiple weigh-ins on the same day Late or insufficient food logging	Educate on scale use Verify and reset weights Confirm food logging
Activity tracker data	Data not received	Fitbit not syncing to phone due to firmware issues or Bluetooth signal issues (Bluetooth off, devices too far away to sync, interference from other sources) Technical bugs with Fitbit app Accidental revocation of authorization Data transmission issues	Manual sync Update firmware Restart Fitbit Restart Bluetooth Increase Bluetooth signal (device proximity/remove interference from other Bluetooth/Wi-Fi signal sources) Reauthorize and send new code

^a^API: application programming interface.

## Discussion

### Overview

Novel strategies are needed to support both the initiation and maintenance of weight loss. Because weight management requires frequent, repeated behaviors, technologies that minimize barriers to tracking may be particularly effective. Mobile technologies allow for continuous monitoring of progress, and with appropriate technical support (Table 1), Log2Lose demonstrates that near real-time, large-scale data collection, and feedback are feasible across time zones and diverse settings. Our experience suggests that automation is scalable when paired with pragmatic, responsive support.

Our findings contribute to the broader literature on incentive-based weight management interventions. Prior studies have shown that financial incentives can support initial weight loss but often face challenges in sustaining long-term adherence [5-7]. More recent work such as Ladapo et al [4] have demonstrated the potential of both outcome-based and behavior-based incentives; yet, questions remain regarding how best to deliver and sustain these approaches in real-world contexts. Log2Lose extends this body of work by demonstrating that incentives can be operationalized at scale through a fully remote, multisite trial supported by consumer technologies. Unlike earlier studies that relied on in-person contact or static incentive structures, our trial highlights the importance of adaptability in the face of shifting technology, regulation, and participant needs. In this way, Log2Lose not only reinforces prior evidence that incentives can be effective but also advances the field by offering a model for integrating automation, flexibility, and responsive technical support into long-term digital health interventions.

### Evolution of Technology

The COVID-19 pandemic necessitated a swift pivot to fully remote delivery. Group weight loss sessions were moved to videoconference, nearly all technical support was provided remotely, and primary outcome data were collected with cellular scales instead of during in-person visits. Preparation during our pilot phase positioned us to navigate an increasingly complex technology and regulatory environment during and after the pandemic [11,20].

To avoid reliance on a single application, the platform was designed to be app-agnostic. This flexibility proved essential when the MyFitnessPal–Fitbit API link ended mid-trial. We migrated dietary logging entirely to Fitbit and routed integrations directly through Log2Lose rather than third-party-to-third-party connections. Although this migration required substantial engineering effort, it improved data integrity and reduced opaque synchronization issues. Later vendor changes such as Fitbit’s consolidation after Google’s acquisition necessitated further protocol revisions and staffing adjustments.

The Federal Communications Commission’s introduction of new text messaging regulations added another layer of complexity. Maintaining message delivery required continual problem identification and resolution, often resembling a game of whack-a-mole, especially for issues that threatened intervention integrity. Some messages, particularly those with repetitive content or dollar amounts, were flagged and blocked by telecom carriers. Weekly incentive messages were not personalized with participant-specific information such as names or pronouns, which may have increased the likelihood of being flagged. Although our platform attempts to resend failed messages automatically, evolving security protocols necessitated manual weekly surveillance. The research team reviewed failures by carrier and content, and small adjustments such as removing numerals often restored successful delivery in subsequent weeks. In today’s rapidly evolving landscape of technology, regulations, and proprietary ownership, adjustments are to be expected in multiyear studies. Building flexibility into planning is therefore essential.

### Automation and Technical Support

Automation processed approximately 99% (126082/126283) of the data and incentives on a fixed weekly schedule, reducing errors and preserving fidelity at scale. Automated systems deliver interventions consistently and without bias, but skilled technical support remains indispensable. Our experience underscores that adequate staffing and technical expertise are critical to the success of large-scale digital health programs.

The platform’s monitoring tools enabled staff to address many technical problems proactively without contacting participants. However, when automated processes failed, whether for email alerts, class reminders, surveys, text messages, or weight data collection, immediate intervention was required. With over 700 participants, resolving issues demanded substantial time and resources. For instance, a scale issue we identified was that placement near a bathroom wall with baseboard molding would create a fulcrum effect, causing inaccurate weight measurements. Daylight savings time caused problems with text message delivery that we did not plan for. In each case, staff investigated root causes through messaging API error codes and implemented targeted solutions to prevent recurrence.

### Limitations

Although more than 85% of US adults own a smartphone [21], generalizability is limited to those with smartphones and data plans and the digital literacy to use the devices, apps, and videoconferencing technology. Message delivery receipts were recorded, but message opens were not tracked because text messaging lacks reliable open tracking functionality. Email open rates were not implemented to protect participant privacy. Data inaccuracies could occur when other household members used the scale, participants underreported dietary intake, or technology literacy was limited. Although such inaccuracies increase variability in efficacy estimates, they are inherent to real-world settings, generalizable to similar interventions, and likely to bias results toward reduced effect sizes rather than inflate estimated effects.

### Conclusions

Log2Lose demonstrated the feasibility of a scalable, digitally enabled weight management program. Success depended on adaptable software built on consumer technology, coupled with continuous monitoring and responsive support. By leveraging widely available consumer technologies, the platform adapted to regulatory and technological changes while maintaining intervention fidelity. Although automation allows for broad reach and efficiency, dedicated staffing for technical support is essential. This program model can inform health care providers, payers, and employers implementing obesity interventions, supporting a healthier workforce and potentially reducing health care costs.

## Appendix

Multimedia Appendix 1 Log2Lose platform population-level dashboards showing (A) messages transmitted in a day with delivery rate and errors; (B) participant information by study site and cohort, connection status, and scale signal and battery; (C) calorie entries, weights, incentives awarded, and messages transmitted; and (D) downloadable reports for study management and analyses.

Multimedia Appendix 2 Log2Lose platform individual participant dashboard example showing calories tracked, weights received, and incentive and text message history.

## Abbreviations

API: application programming interface
REDCap: Research Electronic Data Capture

## Data Availability

Following completion of the Log2Lose trial, a dataset will be publicly available in the Biological Specimen and Data Repository Information Coordinating Center (BioLINCC) [22].
