# Supplementary figures and images for: Development, Challenges, and Evolution of the Log2Lose Intervention for Weight Management: Randomized Controlled Digital Health Trial
Source: JMIR Form Res. 2025 Nov 17;9:e70842. doi: 10.2196/70842 (PMC12670057; doi:10.2196/70842)

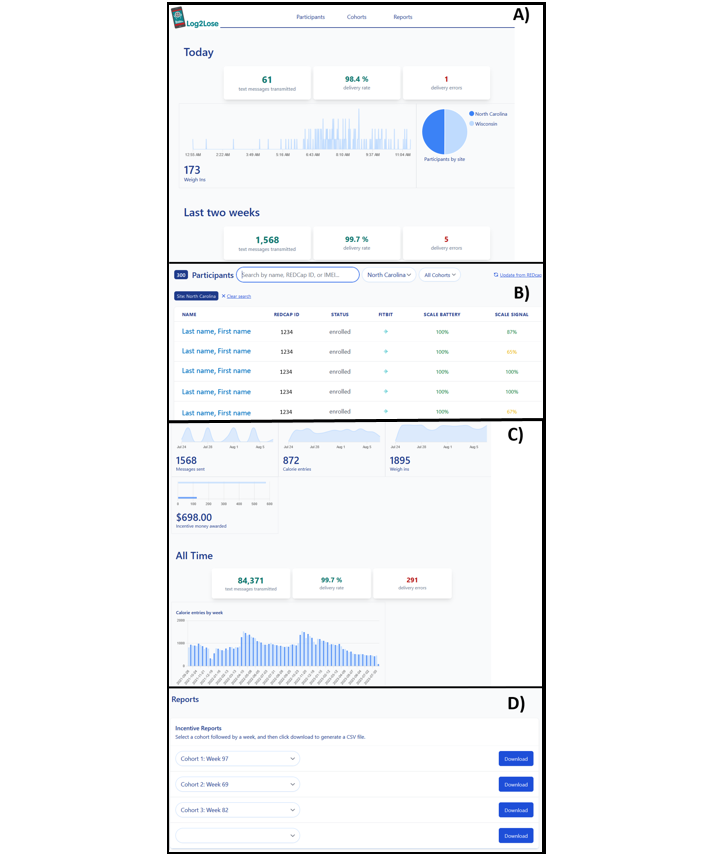

Supplement: Multimedia Appendix 1 [file formative_v9i1e70842_app1.png]

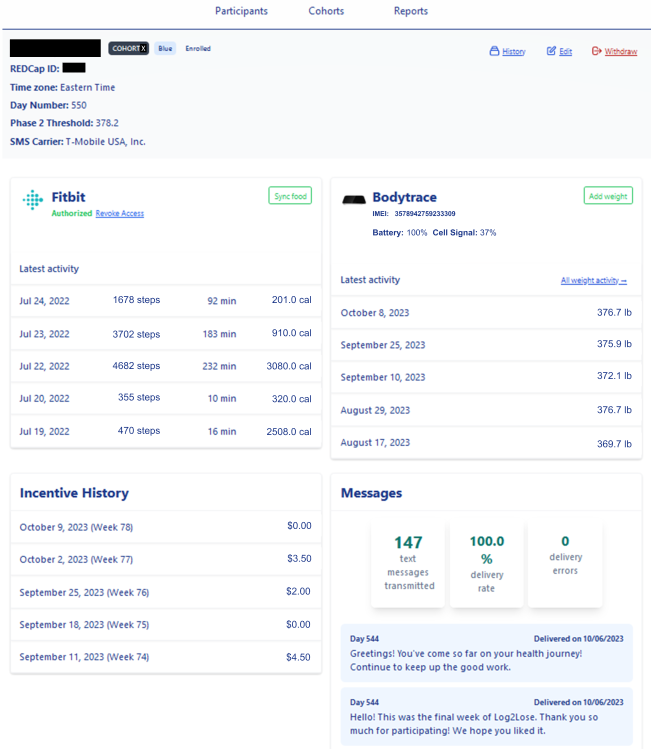

Supplement: Multimedia Appendix 2 [file formative_v9i1e70842_app2.png]
